# Controlling viral inflammatory lesions by rebalancing immune response patterns

**DOI:** 10.3389/fimmu.2023.1257192

**Published:** 2023-08-21

**Authors:** Sachin Mulik, Engin Berber, Sharvan Sehrawat, Barry Tyrrell Rouse

**Affiliations:** ^1^ Center for Biomedical Research, The University of Texas Health Science Center at Tyler, Tyler, TX, United States; ^2^ Infection Biology, Lerner Research Institute, Cleveland Clinic, Cleveland, OH, United States; ^3^ Indian Institute of Science Education and Research, Department of Biological Sciences, Mohali, Punjab, India; ^4^ College of Veterinary Medicine, University of Tennessee, Knoxville, TN, United States

**Keywords:** immunopathology, immunotherapy, immune regulation, immune exhaustion, immunometabolism, microRNA, viral pathogenesis

## Abstract

In this review, we discuss a variety of immune modulating approaches that could be used to counteract tissue-damaging viral immunoinflammatory lesions which typify many chronic viral infections. We make the point that in several viral infections the lesions can be largely the result of one or more aspects of the host response mediating the cell and tissue damage rather than the virus itself being directly responsible. However, within the reactive inflammatory lesions along with the pro-inflammatory participants there are also other aspects of the host response that may be acting to constrain the activity of the damaging components and are contributing to resolution. This scenario should provide the prospect of rebalancing the contributions of different host responses and hence diminish or even fully control the virus-induced lesions. We identify several aspects of the host reactions that influence the pattern of immune responsiveness and describe approaches that have been used successfully, mainly in model systems, to modulate the activity of damaging participants and which has led to lesion control. We emphasize examples where such therapies are, or could be, translated for practical use in the clinic to control inflammatory lesions caused by viral infections.

## Introduction

Since the dawn of the COVID-19 era, persons not claiming to be virologists have learned more facts and fake facts about viruses and how they cause disease than anyone could have imagined. Many of the newly enlightened feel they know so much about COVID-19 to decide that they and their family, including children, do not need to be vaccinated against the COVID-19 virus or even against any infectious agent. This often includes measles, a highly infectious and potentially devastating viral infection of children. However, as all trained medical scientists are well aware, some virus infections, that include COVID-19, can be controlled effectively with vaccines and when this occurs the vaccine approach is more effective, convenient to use and far less expensive than any other viral control measure. Unfortunately, we lack effective vaccines against some virus infections and these need to be controlled by alternative therapies that are often unsatisfactory. In this review, we discuss how we might control virus infections where lesions occur mainly as a consequence of the host`s immune reaction to the infection and make a case for controlling such infections by rebalancing the participation of immune reactants.

Some viruses are endowed with intrinsic pathogenicity and cause clinical disease as a direct consequence of their destroying cells and tissues. In mankind, smallpox was such an example causing marked clinical signs and death in many millions of persons before successful vaccines were discovered and used. When smallpox vaccines became widely available, all were forced, or wisely chose, to use them and in 1979 smallpox was fully controlled and eradicated (1). So far this is the only human virus disease that has been eradicated, but we are, or at least were, close to success with poliovirus (2). We might have succeeded also in eliminating the COVID-19 virus if appropriate public health measures had been adopted early after its initial discovery. Many virus infections are not a major health problem in persons with a normally functioning immune system, but do become so when the activity of one or more aspects of immunity are defective for genetic or other reasons (3, 4). Human cytomegalovirus is such an example with this herpesvirus becoming a significant pathogen in persons that receive immunosuppressive drugs to prevent rejecting their transplants or are co-infected with other agents that suppress immunity, such as untreated human immunodeficiency virus (HIV) infection (5).

**Figure 1:**
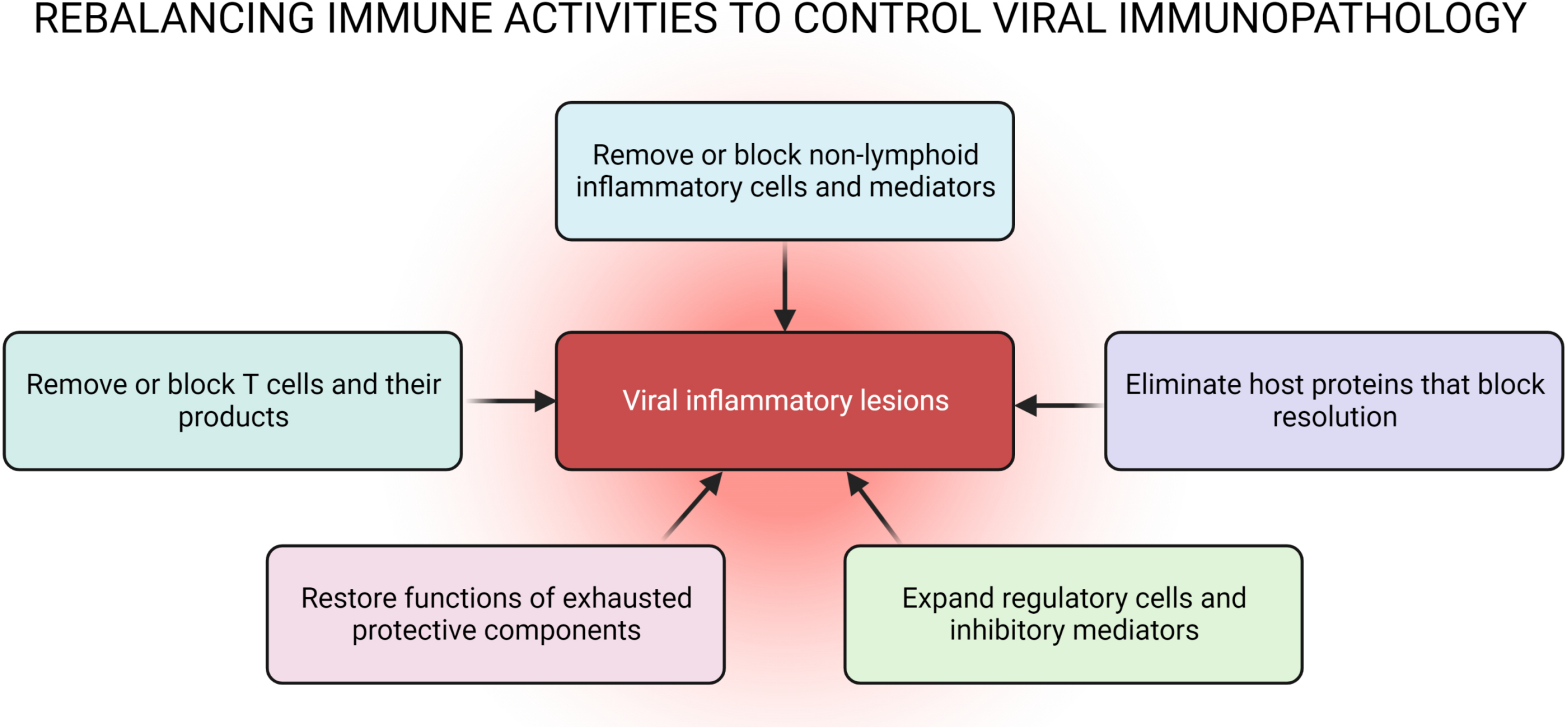
A scheme proposing multiple strategies that have the potential to recalibrate immune activity and reduce the occurrence of lesions. Approaches aimed at inhibiting pro-inflammatory cells and or cytokines, expanding regulatory cells of innate or adaptive immune system, removal of pathogens by reinvigorating exhausted T cells could represent such interventions.

There are also many virus infections which are not cytopathic and fail to cause overt tissue damage when they replicate in cells, but they can elicit tissue-damaging lesions that are often chronic. When this happens, usually the lesions are largely the consequence of the host`s response to the infection (3). As immunologists have taught us, the host can respond to invaders in many ways and only some of these aspects may be responsible for causing the pro-inflammatory effects. In the case of viral immunoinflammatory lesions, the pro-inflammatory participants are usually a subset of T cells along with nonlymphoid inflammatory cells (6, 7). At the same time other aspects of the host`s innate or adaptive immunity also may be engaged with these acting to diminish the extent of damage and contributing to resolution. Given such a scenario the case can be made that if ways are used that rebalance the participation of the damaging and protective components to favor the latter then the impact of viral inflammatory lesions will be diminished. In this review, we describe several ways that can achieve a rebalance of immune responsiveness and emphasize those approaches that might be most practical for use in the case of human tissue-damaging viral infections. A scheme defining several strategies that could rebalance immune activity and diminish lesions is depicted in **Figure 1**.

## Minimizing inflammatory reactions by rebalancing aspects of the innate immune response

Innate immunity constitutes a first line of defense against invading pathogens. The system is set into action by pathogen-associated molecular patterns (PAMPs) present within or on the surface of pathogens or generated from tissues damaged by the pathogens–so-called damage associated molecular patterns (DAMPs) (8). These PAMPs and DAMPs are sensed by several types of pattern recognition receptors (PRR) expressed by the multiple types of innate immune cells. The responding cells undergo a range of changes that include migratory activity, activation, metabolism status, morphological alterations, acquisition of molecules responsible for their migratory activity, as well as the production of several molecules that can influence the function of other cell types, such as those that constitute the adaptive immune system (9, 10). There are multiple types of innate immune cells and these include dendritic cells (DC), macrophages, natural killer (NK) cells, granulocytes, gamma delta T cells and innate lymphoid cells and all cell types could influence in some way the outcome of a viral infection. However, perhaps the most relevant first responders to viral infections are DC, which are themselves quite heterogeneous in terms of the actual PRR they mainly express, as well as the major molecules they produce once stimulated by PAMPs or DAMPs (11, 12). Moreover, DC are superior antigen presenting cells and this function, along with molecules produced such as cytokines, results in a variable outcome in terms of the quantity and quality of the subsequent adaptive immune response induced (13, 14). Notable DC products also include interferons, which in addition to having antiviral activity also help shape the nature of immune responses to infection (15). Given this heterogeneity of the first responders, there is clearly an opportunity to introduce modulators, particularly early during the initial infection process that will favor the engagement and activation of some subsets of innate cells over others (16). Manipulating innate aspects of immunity to improve immunity has been exploited for decades by formulating vaccines that include adjuvants that improve immunity by acting on one or more aspects of innate immunity (17). Manipulation of innate immune function could, for example, diminish the induction of pro-inflammatory tissue-damaging responses favoring the induction of regulatory responses and anti-inflammatory cytokines. However, once the pattern of immune responsiveness is established after immune induction, changing this pattern by manipulating innate cell composition and their functions becomes far less accessible and this is the challenge we are discussing in this review.

**Figure 2:**
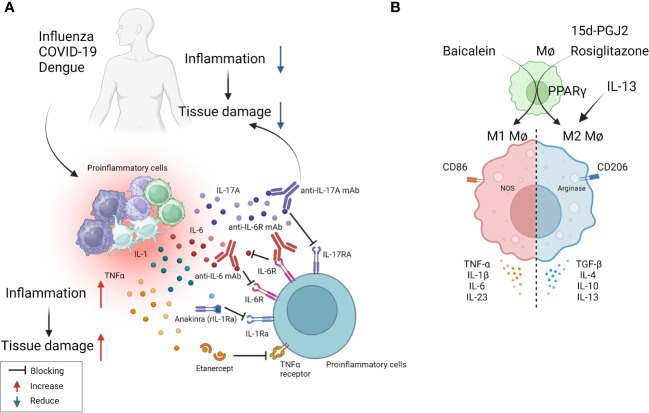
Some approaches tested clinically **(A)** and in in-vivo model systems **(B)** to resolve inflammation. **(A)** Multiple virus infections are presented with a surge in cytokine levels contributing to inflammation and tissue damage. Blocking the activity of IL-1, IL-6, IL-17, TNF-α conferred therapeutic benefit in COVID-19 patients and is likely useful in other viral pathologies involving cytokine storms (anti-IL-6 mAb: Siltuximab, anti-IL-6R mAb: Sarilumab and tocilizumab, anti-IL-17A: Netakimab). **(B)** M1 or M2 macrophages differentially contribute to tissue damage after virus infection. Activation of PPARγ with 15d-PGJ2 or rosiglitazone and resultant M2 cell expansion led to diminished lung pathology after influenza and RSV infection respectively. Contrary results also exist, as M1 cell expansion using Baicalein led to diminished inflammatory response to influenza. Thus, inducing polarization of macrophages can recalibrate immune response after virus infection.

The strategies available to effect changes in innate cell activity can exploit the fact that the different innate cells express a diverse array of PRR that respond to the many PAMPs expressed by the infecting agent and DAMPs resulting from tissue damage. Accordingly, by using PRR ligands or inhibitors it should be possible to change the participation of various innate cells (18). In addition, innate modulations could target signaling events set into motion as well as the protein products of innate cells that mediate their influence. With regard to manipulating the diverse array of PRRs, some key ones that mold the pattern of innate responsiveness to viruses are the multiple Toll-like receptors (TLR) that are expressed either at the surface or within cells that compose the innate immune system (10). Others include the melanoma differentiation-associated protein 5 (19), retinoic acid-inducible gene I receptors (20), nucleotide oligomerization domain, as well as receptors which recognize complement components and their breakdown products (21). There are additional nucleotide sensors in the cytoplasm such as cyclic GMP-AMP synthase, which senses DNA derived from viruses (22). A list of innate sensors and viral ligands is provided in **Table 1**. For instance, TLR-2, TLR-4 are triggered by some viral envelope or capsid components (47–49). TLR-3 senses double stranded RNA which many viruses produce during their replication (50). TLR-7 and TLR-8 sense single-stranded RNA (51) while viral DNA is sensed by TLR-9 (27, 52). These TLRs are differentially expressed on innate cells setting the stage for a virus expressing one or more PAMPs triggering some, but not all, innate cells depending on their PRR expression. Moreover, the innate cell triggering may lead to a cascade of signaling events and the production of cytokines and chemokines that in turn mediate cell recruitment and aspects of the tissue-damaging inflammatory reaction. Consequently, strategies available to rebalance the participation of innate immune mechanisms, are available and some these are listed in **Figure 2** and are discussed below.

**Table 1:** Examples of several innate immune sensors that recognize viral ligands.

Activation ligand	Viruses recognized by innate sensors	Innate immune sensor	Ref
Envelope protein, RNA	Influenza A virus, HIV	TLR10	(23–26)
CpG-DNA motifs	HSV-1, HSV-2, MCMV, Poxvirus, Adenovirus, Polyomavirus	TLR9	(27–29)
Glycoprotein, envelope protein, fusion protein	Respiratory syncytial virus, Ebola virus, Human immunodeficiency virus	TLR4	(30)
Envelope glycoprotein, core protein	HSV-1, Measles virus, Hepatitis C virus	TLR2	(31–33)
dsDNA	HSV-1, Dengue virus, HIV-1, Adenovirus	cGAS	(34–37)
dsRNA, Circular-RNA, ssRNA,	Dengue virus, Influenza A virus, Japanese encephalitis virus, Rift valley fever virus	RIG-I	(19, 38, 39)
dsRNA, RNA	West Nile virus, Measles virus, Rotavirus	MDA5	(40–42)
M2 protein, viral RNA	Influenza A virus (IAV)	NLRP3 inflammasome	(43, 44)
Cytoplasmic DNA	MCMV, Vaccinia virus	AIM2 inflammasome	(45)
Single-stranded RNA	Vesicular stomatitis virus, RSV, IAV, parainfluenza virus 3	NOD2	(46)

In the majority of viral immunopathological reactions, the lesions are orchestrated by adaptive immune components, usually T cells, but the actual tissue damage is caused mainly by nonlymphoid inflammatory cells and several inflammatory mediators released from activated innate as well as adaptive cell types (3). These mediators often constitute what is called a cytokine storm (53). Prominent among the cellular participants are macrophages, particularly those described as M1 macrophages. Thus, lesion severity can be diminished by removing macrophages or by changing the balance of macrophage subtypes from a dominance of M1 to the M2 subset, more often involved in lesion resolution. There are approaches to counteract macrophages and their activities and these have been used in numerous model systems of viral immunopathology. The initial approach was developed by Nico Van Rooijen and involved using clodronate liposomes that destroyed cells, predominantly macrophages that phagocytosed the liposomes. The clodronate, once in the cytoplasm, is metabolized by aminoacyl t-RNA synthetases to generate an ATP analogue that in turn trans locates to the mitochondrial membrane, inhibiting mitochondrial ATP/ADP translocase. This results in inhibition of mitochondrial respiration, as well as the induction of apoptosis (54, 55). This approach has been used to define the role of macrophages in several viral infections in model systems. These include respiratory disease caused by pneumovirus and the neuropathological consequences of Theiler's virus infection in mice (56, 57). Macrophage depletion was also reported to attenuate muscle and joint inflammation after Ross River virus infection in mice (58). Furthermore, clodronate liposomes given intranasally result in less severe lesions in mice infected with respiratory syncytial virus (RSV) (59). All of these studies were interpreted to mean that macrophages were involved in causing the tissue damage at the overt lesion stage, but the experimental design invariably involved depleting macrophages from early stages of infection, so could not exclude the influence the cells also might be having during immune induction or reveal how macrophages participate in inflammatory reactions. As far as we know, human viral immunoinflammatory lesions have not been managed using the clodronate approach.

In more recent times, it has become evident that macrophages can polarize into pro-inflammatory M1 cells mainly involved in mediating tissue damage and M2 cells that largely play an anti-inflammatory and tissue repairing function. The M1/M2 concept was introduced by Mills and came from experiments showing that macrophages from Th1-prone mouse strains (such as C57BL/6 and B10D2) exhibit a strong nitric oxide response against stimulants such as LPS, while macrophages from Th2-prone strains (such as BALB/c and DBA/2) exhibited a strong arginine metabolism response, an effect not observed in macrophages from Th1 prone mice (60). These two functional subtypes can readily be differentiated from precursors in vitro and shown to differ in the types of cytokines they can produce, surface characteristics, and the expression of some critical enzymes involved in their function. For instance, M1 cells are CD86 positive and produce nitric oxide synthase and reactive oxygen products. They mainly produce the cytokines TNF-α, IL-1β, IL-6, and IL-23. M2 cells are CD206 positive, arginase positive, and mainly produce the cytokines IL-4, IL-13, IL-10, and TGF-β (61). Whereas there are no convenient ways in vivo, especially in humans, to selectively deplete the subtypes and demonstrate their role in tissue damage, in model systems it is feasible to preferentially expand the different cell types and record any change in the expression of a virus infection. Additionally, studies can be done where mice are manipulated to generate predominantly M1 or M2 macrophages and then to compare the outcome of a viral infection. Using the latter approach one group has implicated that M1 cells are more involved than are M2 cells in ocular inflammatory lesions caused by herpes simplex virus (HSV) infection (62). Others reported that M1 macrophage polarization occurs in the brain after multiple flavivirus infections, and that inhibiting this polarization by blockading the M1 cytokine product TNF-α significantly attenuated Dengue virus-induced neurotoxicity, a lesion that involves a host inflammatory reaction to the virus (63). However, it is not clear if polarization changes can be accomplished once inflammatory lesions have commenced and are ongoing, as would be needed in clinical situations.

Another procedure that has been used to switch the balance of M1 and M2 macrophages is to use agonists of the transcription factor peroxisome proliferator-activated receptor gamma (PPAR-γ), which is a member of the nuclear receptor family and is involved in regulating several inflammatory genes (64). It was shown that the interaction between the signal transducer and activator of transcription 6, promotes macrophage polarization towards to the anti-inflammatory M2 type along with the expression of IL-4 and IL-13 (65). For example, using mice infected with RSV and then treated with the PPAR-γ agonist pioglitazone from day 2, resulted in diminished lung pathology explained by M2 cells dominating the lesions rather than M1 cells as is the case in lesions (66). Unfortunately, the majority of these macrophage subtype rebalancing therapies were started in the immune induction phase and continued on rather than addressing the issue we are discussing of making changes in established lesions to alleviate their severity. To this point therapy with the PPAR-γ activator, 15d-PGJ2, resulted in significantly reduced lung inflammatory reactions and mortality to influenza (FLU) infection, but only if therapy was begun on day 1 and not when lesions were already present (67). Thus, the PPAR-γ agonist approach may have minimal value to control viral inflammatory lesions once they are already underway. Additional comments about PPAR-γ are made in a subsequent section since PPAR-γ impacts on some genes involved in metabolic pathways.

Although the idea that controlling viral inflammatory lesions can be achieved by approaches that suppress M1 or enhance M2 macrophages is a useful notion, it has yet to become an accepted paradigm. Indeed, contrary data for the concept does exist. For example, there are reports that expanding the M1 population, as can be achieved using the molecule Baicalein, causes a diminution of inflammatory responses to FLU infection (68). In addition, whereas macrophages, particularly M1 cells, may directly participate in causing tissue damage, the cells may also participate indirectly to mediate inflammatory effects by releasing soluble mediators such as TNF-α (69). Overall, macrophage-targeted approaches hold promise to control inflammatory viral lesions, although achieving such control in practical situations such as in a chronic human viral infection still needs to be realized.

As mentioned previously, tissue damage is often caused by inflammatory cytokines and chemokines which can be the products of innate immune cells, although some of the cytokines also may be the products of adaptive immune cells. In several viral infections, where the host response is a major contributor to the tissue damage; multiple cytokines and chemokines can be involved constituting what is usually referred to as a cytokine storm (53). Such storms are a major feature of severe Dengue viral infections, but also occur in some patients with severe COVID-19 lesions as well as occasionally in FLU (53, 70). Controlling cytokine storms therapeutically is currently achieved using anti-inflammatory drugs, but monoclonal antibodies (mAbs) against some pro-inflammatory cytokines, or their receptors, also can be effective therapies. For example, severe COVID-19 patients often experience a cytokine storm that includes multiple cytokines (71). A commonly used control measure is to target some of these, but especially IL-6 and its receptors, with a mAb to control the severe inflammatory lesions so preventing multiorgan failure and aiding recovery (72). Accordingly, clinical trials showed that anti-IL-6 receptor mAb (e.g., sarilumab, tocilizumab) and anti-IL-6 mAb (e.g., siltuximab) reduced inflammation, decreased the need for mechanical ventilation, and resulted in a 45% reduction in death in COVID-19 cases (72). As a result, tocilizumab has been included in some treatment guidelines for severe COVID-19. Additionally, Anakinra, a recombinant IL-1 receptor antagonist, also was suggested as a potential treatment for the hyperinflammatory state linked to SARS-CoV-2. Accordingly, a study with 52 patients found that subcutaneous Anakinra treatment reduced the need for mechanical ventilation in the ICU and decreased mortality rates in severe COVID-19 patients without significant side effects (73). However, further controlled trials are necessary to confirm the effectiveness of Anakinra. Similarly, in severe Dengue virus inflammatory disease, there is significant upregulation of multiple cytokines compared to healthy controls and lesions can be diminished by blocking some of these with specific mAb (63, 74). In H5N1 FLU, patients show elevated levels of IL-6, IL-8, IL-1β, IFN-γ, TNF-α, and the soluble IL-2 receptor, but the outcome of blocking one or more of these cytokines needs to be evaluated in humans. However, when tested in mice, blockade of TNF-α, IL-1β attenuated lung pathology after FLU infection of mice (75, 76). Targeting some chemokines is also a useful way to diminish the consequence of inflammatory viral lesions. In a study using a chronic obstructive pulmonary disease-induced mouse model caused by H1N1 FLU infection, treatment with the CCR5 chemokine antagonist, maraviroc, led to a significant reduction in lung pathology with concomitant reduction in the numbers of infiltrating neutrophils and macrophages in lung airways, as well as increased survival of mice (77). In the context of COVID-19 infection, terminally ill patients showed restored plasma IL-6, CD4, and CD8 responses after receiving two doses of leronlimab, a CCR5-specific IgG4 mAb, in the ICU while on mechanical ventilation. Although lacking a control group, the results suggest that anti-CCR5 treatment significantly reduced inflammatory reactions compared to baseline in plasma samples, which might have prevented pulmonary pro-inflammatory leukocyte infiltration (78). Supporting these findings, a clinical trial registry (NCT04347239) comparing anti-CCR5 mAb treatment with dexamethasone as standard care or placebo resulted in significantly increased survival (79). Along a similar line, in ferret models for FLU, inhibiting CXCL10 activity with the CXCR3 inhibitor, AMG487, which blocks its signaling, increased survival length and diminished lung pathology (79). The study also reported a reduction in viral load in the lungs, which might be correlated with the establishment of effective antiviral responses in H5N1 infected and CXCR3 antagonist drug-treated ferrets (80). Other research has tested blocking CCR2 using the inhibitor, PF-04178903, in mice infected with H1N1 FLU. The results showed that CCR2 blockade reduced mortality and clinical implications without altering viral titers, suggesting that CCR2 antagonists could potentially serve as an effective therapy against FLU-induced pathogenesis (81).

In this section, we have evaluated the prospect of managing viral inflammatory lesions by changing the function of innate aspects of immunity. Few approaches that target and change aspects of innate immunity can be applied in practical situations, but strategies have succeeded in model systems, although they are less effective when used to counteract already established lesions. The most effective practical therapies are those that counteract inflammatory cytokines and chemokines most of which are proprietary humanized mAbs and so are very expensive to use. Nevertheless, some have been valuable therapies to control inflammatory lesions in COVID-19 patients as well as in severe Dengue. Conceivably, lesion control also could be achieved by administering anti-inflammatory cytokines such as IL-10 and TGF-β, but they have a short half-life and might be more effective if delivered directly to inflammatory lesions. Conceivable problems with delivering cytokines could be overcome by using half-life extended fusion cytokine proteins since this approach has been successful in mice using fusion proteins that deliver IL-10 to treat solid tumors (82). We anticipate that chemical reagents that can change the functional type of macrophages in lesions could be in the pipeline, as could be ligands that act on TLRs and inflammasomes. For example, the TLR7 agonist, Imiquimod is dispensed topically to treat genital warts caused by papillomavirus infection (83). In addition, several mouse models have shown the value of using approaches that target TLRs and inflammasomes (84–87), although few if any studies start therapy when significant lesions were already present.

## Minimizing inflammatory reactions by manipulating the activity of adaptive immune participants

The idea that lesions manifest in some viral infections represent immunopathological reactions arose largely from pathogenesis studies on the non-cytopathic virus Lymphocytic choriomeningitis virus (LCMV) by Mims and Blanden >50 years ago. As another pioneer in the LCMV field, Michael Oldstone, liked to claim all major mechanistic discoveries in viral pathogenesis and many in immunobiology in general came from studies using LCMV (88). With this infection, the choriomenigitis is a consequence of immunopathology with CD8 T cells orchestrating the lesions and the glomerulonephritis that often occurs is a lesion resulting from the trapping of immune complexes that cause an inflammatory reaction (89), a topic rarely studied by contemporary investigators. Furthermore, studies using LCMV revealed how T cells recognize antigens for which Doherty and Zinkernagel were awarded the Nobel prize in 1996 (90). The idea that all instances of viral immunopathology involved CD8 T cells gained gravity, but it seems likely that in human viral immunopathologies either CD4+ Th1 or Th17 T cells are more often involved in directing the inflammatory lesions. Studies on LCMV clearly showed CD8 cells cause tissue damage mainly by directly destroying infected cells, but when CD4 cells are the orchestrators the tissue damage is usually indirect and involves the release of mediators that recruit a range of nonlymphoid pro-inflammatory cells and tissue-damaging activities (89). In both situations, controlling the lesion severity should occur if the orchestrating T cells are removed, or their function and products inhibited, such as by cells or proteins with regulatory function as is discussed in a later section. Therapies directed against T cell orchestrators of lesions have proven successful in many mouse model systems of viral immunopathology. These include HSV induced stromal keratitis used by our group (91), Theiler's virus-induced neuropathology (92), Coxsackie virus-induced myocarditis (93), West Nile fever virus lesions (94) and several others (95, 96). However, in the models, a wide range of sophisticated approaches can be used. These include several in vivo genetically engineered systems that can directly implicate one or another cell type, numerous specific mAbs that block cells or cytokines and their receptors, adoptive cell transfer strategies with intact and gene modified cells along with some drugs and small molecule inhibitors that selectively block the activity of a particular pro-inflammatory cell type. We will not review these many observations made in model systems since most of the experimental maneuvers could not be applied to rebalance and control a clinical situation of viral immunopathology. However, in **Table 2** we list some general approaches used in model systems that have succeeded in defining the participation of critical cell types and their products that mediate immunopathology. In addition, many small uncontrolled therapies directed at T cells and their products have been explored to limit the inflammatory stage of COVID-19. For example, a retrospective study blocking Th17 T cells with the anti-IL-17 mAb netakimab proved effective and was well tolerated (101). In addition, a trial in Bangladesh that combined an anti-IL17A mAb with the JAK inhibitor Barictib was effective against COVID-19 respiratory disease, although the therapy was followed by a higher frequency of secondary infections than in controls (102). Moreover, as discussed in the previous section, mitigation of the severity of COVID-19 lesions has also been achieved using mAbs against some cytokines produced by inflammatory T cells as well as by innate cell types.

**Table 2:** Some approaches used to control inflammatory lesions that target adaptive immune participants.

Approaches	Outcome observed in vivo	Ref
Use of genetically engineered mice where specific cell types can be deleted, or their function blocked	Deficiency of IL-17 receptor mitigated HSV-1 induced immunopathology in mice	(97)
	Absence of IL-17A inhibits EAE development	(98)
	Lack of IL-17A in CD4 T cells attenuated development of EAE in mice	(99)
	RORgT deficiency in T cells results in absence of tissue infiltrating Th17 cells and inhibits EAE development	(100)
Use of mAbs with or without Toxin Expression	Neutralization of IL-17A diminished HSV-1 induced corneal immunopathology in mice	(97)
	Blocking IL-17A during induction phase attenuated EAE disease in mice	(98)
	Blocking IL-17A was beneficial in COVID-19 patients	(101, 102)
	Blocking lymphotoxin alpha inhibited Th1 and Th17 cells and mitigated ocular lesions after HSV-1 infection	(103)
Using small molecule inhibitors that block the function of specific cell types	Small molecule inhibitors can selectively block the activity of Th17 cell types involved in inflammatory lesions, suppressing the production of pro-inflammatory molecules and reducing inflammation.	(104–106)
Genetic modification of adaptive immune cells and adoptive cell transfers	Adaptive immune cells are modified by CRISPR/Cas9 or CAR technology and gene modified T cells are transferred into mice to attenuate inflammatory damage.	(107, 108)
Use of antagomirs, siRNAs or methods to target genes in Th17 cells	Antagomir sequences, siRNAs or LNA are used to target genes in Th17 cells and to mitigate inflammation.	(109–113)
Use of epigenetically acting drugs to mitigate inflammatory damage	Epigenetically acting drugs are used that inhibit inflammatory adaptive immune cells or to increase regulatory cells	(114–117)
Using T cell exhaustion/check point therapy to reversing T cell exhaustion, thereby enhancing the immune response to clear the infection.	PD-1 blockade was effective on CXCR5+ progenitor Tex, inducing proliferation and cytokine production to clear LCMV infection, but was without effect on terminal Tex	(118)
	Dual blockade of Tim-3 and PD-1 or combining PD-1 blockade with IL-2R agonist substantially enhanced virus-specific CD8 T cell responses, increasing the numbers and cytokine production compared to single checkpoint blockade in LCMV infection	(119–121)
	The combination of PD-L1 blockade with 4-1BB costimulation led to significantly improved antiviral CD8 T cell responses in chronic LCMV infection	(122)

There are some general approaches that have achieved efficacy in model systems that could be translatable. One is to use small molecule inhibitors that can specifically target pro-inflammatory T cells and disarm their function (104–106). For example, the small molecules CQMU151 and CQMU152 target the transcription factor RORγt needed for pro-inflammatory Th17 T cells to function (106). Furthermore, therapeutic administration of CQMU151 and CQMU152 attenuated the clinical severity of experimental autoimmune uveitis, experimental autoimmune encephalomyelitis and type 1 diabetes in mice (106). However, the drug has not been tested as a means to counter ongoing viral immunopathology. There are other druggable targets of T cells that might translate from successful results in model systems. For example, treating ongoing lesions of herpetic ocular lesions with the DNA methyltransferase inhibitor 5-azacytidine diminished lesions, although the effect was more to expand the suppressive function of regulatory T cells (Treg) than being inhibitory to pro-inflammatory T cells (123). Also of potential value are antibiotics derived from streptomyces that were shown to inhibit ongoing autoimmune lesions mediated by Th17 T cells (124). Another strategy is to target molecules such as lymphotoxin alpha expressed by Th1 and Th17 (125). It was shown that therapeutic administration of a lymphotoxin alpha blocking antibody given after infection attenuated the severity of ocular lesions (126). Similarly, therapeutic administration of the drug 2, 3, 7, 8- Tetrachlorodibenzo-p-dioxin, which activates the transcription factor aryl hydrocarbon receptor and inhibits Th1 and Th17 cells, but also expanded T cells with regulatory activity, diminished the severity of herpetic ocular lesions (103), an example of a successful immune rebalancing scenario. There are reports also of elevated levels of immune complexes during chronic LCMV infection with such immune complexes impairing otherwise protective antibody effector functions mediated by Fcγ-receptor (FcγR) activity (127, 128). This raises a challenge in chronic virus infections for testing antibodies whose effect is FcγR dependent.

Another perhaps longshot approach to rebalance and control a viral inflammatory reaction came from the Iwasaki lab (129). They advocated a so-called prime and pull approach to control HSV inflammatory reactions in the genital tract (129). Priming meant virus immunization in their model animal system and pull meant subsequently using chemokines to attract immune T cells to the infection site to resolve the lesions. Extensions of this idea have used more acceptable pulling agents such as the non-toxic aminoglycoside antibiotic, neomycin (130). This idea was also advocated for use to counteract human genital HSV lesions where persons are already lifelong latently HSV-infected and hence primed. The pulling agent was advocated to be topical application of the TLR-7 agonist, Imiquimod, an approach shown to be effective in a guinea pig model (131) with Imiquimod approved at least for external topic treatment of human warts (83). Conceivably, the prime and pull approach may be tried in the clinic to control troublesome recurrent herpetic inflammatory lesions.

Finally, an approach to rebalance the role of adaptive immune cells in an inflammatory viral infection, is to manipulate the composition of the microbiome at surface sites. Thus, largely from studies done on controlling autoimmunity, it has become evident that the composition of the microbiome, particularly in the intestinal tract, can influence the extent of inflammatory responses mediated by T cells (132). Accordingly, the dominance of certain microbes will favor the systemic induction of Th17 T cells and hence increase the incidence and severity of some AIDs and likely too of viral inflammatory lesions (100). However, the predominance of other microbes favors the induction of regulatory T cells, which can suppress inflammatory reactions (133). Manipulating the microbiome composition, which can be achieved most conveniently by dietary measures, holds high promise to modulate the development and severity of immunoinflammatory diseases. Unfortunately, the approach has more value to prevent an inflammatory problem than being an effective way to manage established chronic lesions. More mention of this topic is made in the section discussing metabolism.

This section reviewed approaches targeting adaptive immune components to rebalance immune responsiveness so as to mitigate virus-induced tissue damage. Numerous strategies showed value when tested in model systems, but in most cases these studies were done in a way that would not meet the challenge of rebalancing the pattern of immune responsiveness in an established clinical situation in humans or companion animals. However, control of the immunopathological stage of COVID-19 with mAbs to T cell subsets and cytokines has shown promise, but clearly more translational research is merited to develop practical approaches to rebalance the participation of adaptive immune participants to curtail the consequences of viral immunopathology.

## Rebalancing reactions by expanding the activity of regulatory mechanisms

The late and much missed Dick Gershon popularized the concept in the 70s that suppressor cells could put a break on immune responses and constrain their over-reaction (134). Suppressor cells faded from fashion largely due to their unambiguous identification. However, the idea came back with a vengeance in the late 90s when such markers were discovered by groups at NIH and in Japan and the cells were renamed regulatory cells, which were T lymphocytes (135, 136). The regulatory T cells (Treg) were shown to express CD4 and a high affinity subunit of IL-2 receptor (alpha chain also known as CD25) on their surface and they accounted for around 5-10% of the total CD4+ T cells in naïve mice, as well as healthy humans (135). Subsequently, a more reliable identifier, the transcription factor FoxP3 that controls some of the regulatory activities, was discovered (137). With the description of a canonical transcription factor driving the differentiation and function of these cells, their further genetic, molecular, and biochemical analysis became possible, and this led many to believe that such cells could be used for managing inflammatory conditions. Whereas FoxP3+ Treg remain the vanilla flavor, as Shevach later described (138), we now have almost as many flavors of regulatory cells as we have ice creams in parlors. In addition to the activity of regulatory cells, the extent of immune reactions can be limited by several inhibitory cytokines, in particular IL-10, TGF-β and IL-35 (139, 140). One well studied type of regulatory T cells, so-called Tr1 cells, are FoxP3 negative and produce the anti-inflammatory cytokine IL-10 (141). Of its several activities, IL-10 can downregulate class II MHC and can also interfere with the NF-kB pathway to affect immunosuppressive functions (141). Unlike FoxP3+ Treg, such cells may not require physical contact with the effectors to cause immunosuppression. Regulatory cells can also have innate immune features that mainly function during the early phase of viral encounter, or be adaptive antigen specific cells relevant in controlling excessive inflammatory reactions.

When Treg became an accepted part of immunobiology, the focus was on their role in constraining autoimmune lesions and this role could be firmly established following identification of FoxP3 as their canonical transcription factor. Thus, naturally occurring genetic defects in FoxP3 as was observed in scurfy mice as well as in humans with the Immunodysregulation polyendocrinopathy enteropathy X-linked syndrome (142). In addition, when FoxP3 was removed as could be done in model systems either by gene knockout approach or a conditional deletion by diphtheria toxin in transgenic FoxP3-DTR mice, multiple inflammatory and autoimmune diseases resulted (142, 143). Subsequently, it became evident that the function of Treg was involved also in controlling the extent of inflammatory reactions in viral infections (see **Table 3**) and that their over activity could be detrimental in many cancers (156). With respect to chronic viral infections, many studies demonstrated that lesions become more severe if cells with regulatory activity, most commonly CD4+FoxP3+ T cells, were absent or depleted (146). Our own laboratory made the initial observations for a viral infection by showing that immunity to HSV was influenced by Treg and subsequently that the severity of ocular inflammatory lesions caused by HSV were more damaging if Treg were depleted (144, 146). Results with other viral inflammatory lesions told a similar story, but FoxP3+ Treg were not the only cell type involved in limiting the inflammatory reactions (157). In some viral inflammatory lesions, the regulatory cells were identified as CD8+ T cells and in others termed Tr1 CD4+ T cells that produce IL-10 (158). In addition, the FoxP3+ Treg are themselves heterogeneous falling into two major categories; so-called natural Treg (nTreg) that derive from the thymus and mainly react with self-antigens and induced Treg (iTreg) that recognize exogenous antigens, such as viral antigens. The latter group are those mostly involved in limiting inflammatory responses to viruses, but these too are heterogenous some expressing the transcription factor Tbet and are critically involved in regulating Th1 effector cells (159). Acquisition of a phenotype by Treg similar to that of effectors of different helper subsets was also demonstrated, but why it is critical to exert potent suppression is not known (160). A common feature of iTreg is that they can be plastic, losing their regulatory function and even converting to become pro-inflammatory, an event more likely to occur in an inflammatory environment. Hence, the challenge to therapy is both to find ways of expanding the representation of cells with regulatory activity and also to maintain the function of those cells already expressing regulatory function. The issue is how do we achieve these objectives, and can it be done in clinical situations, such as when viral induced inflammatory lesions are already present?

**Table 3:** Some Strategies that target Treg cells and outcomes observed following use in-vivo.

Studies targeting Treg cells	Outcomes observed in-vivo	Ref
Adoptive transfer of CD4+CD25+ cells	CD4+CD25+ cell depletion prevented autoimmune disease development in mice	(135)
Depletion of CD25+ Treg cells in mice using anti-CD25 treatment before infection	The numbers and functionality of HSV-1 specific CD8 T cells enhanced	(144)
Suppression of Treg cells by in vivo treatment with anti-GITR during persistent infection with Friend virus	Improved the secretion of IFNγ from adoptively transferred CD8+ T cells and diminished viral titers	(145)
Adoptive transfer of CD4^+^CD25^+^ T reg cells to SCID mice	CD4^+^CD25^+^ Treg cells attenuated the immunopathological lesion severity on the eye in HSV-1 infected SCID mice.	(146)
CD4+CD25+ cells were depleted by negative selection from peripheral blood mononuclear cells (PBMCs) obtained from humans infected with the HCV.	In peripheral blood, depletion of CD4+CD25+ cells led to the expansion of hepatitis C virus-specific IFN-γ expressing CD4+ and CD8+ T cells in vitro.	(147)
Rapamycin induced CD4+CD25+FoxP3+ Treg cells in-vitro and adoptively transfer to allograft recipients	Rapamycin induced Treg cells prevented allograft rejection in allogeneic pancreatic islets recipient mice.	(148)
Treg cells depletion in DEREG mice with diphtheria toxin before West Nile virus infection	Treg depleted mice experienced more severe symptoms and lethality compared to Treg intact mice.	(149)
Using DEREG mice model to deplete Treg in experimental allergic airway inflammation	Depletion of Treg cells in experimental allergic mice study resulted in exacerbation of airway inflammation	(150)
Depletion or boosting Treg cells during experimental RSV infection in DEREG mice	Treg depletion in mice before RSV infection led to increase in disease severity and inflammatory cellular response in lungs. However, enhancing Treg numbers via IL-2/anti-IL-2 complexes attenuated disease severity.	(151)
Treg cells transiently expanded in mice using anti-CD28 mAb or Treg cells depleted in mice using DEREG model to study Measles viral persistence in CNS	Treg depletion in DEREG mice enhanced the virus specific CD8+ effector T cells in CNS while Treg expansion resulted in virus spread to the brain.	(152)
Depletion of Treg cells in DEREG mice using diphtheria toxin	Depletion of Treg cells resulted in severe corneal lesions in ocular HSV-1 infected mice.	(140)
Depletion of Tregs in Balb/c mice model before RSV infection using anti-CD25 mAb treatment	Treg depleted DEREG mice revealed delayed viral clearance and severe disease when compared to Treg intact mice.	(153)
Use of the DNA methyltransferase inhibitor 5-azacytidine to improve the stability and performance of regulatory T cells	Administration of azacytidine to HSV-1 infected mice starting from five days post infection improved the suppressive activity of Treg cells and diminished ocular lesion immunopathology	(114)
Stabilization of Treg cells using retinoic acid treatment	Retinoic acid treatment diminished lesion severity and reduced the numbers of inflammatory cells in the eyes of HSV-1 infected mice	(154)
Dietary supplementation of short chain fatty acid (SCFA) into drinking water to induce Treg expansion	SCFA supplementation reduced the inflammatory immune response in the corneas and prevented the development of herpetic lesions after HSV-1 infection.	(155)

**Figure 3:**
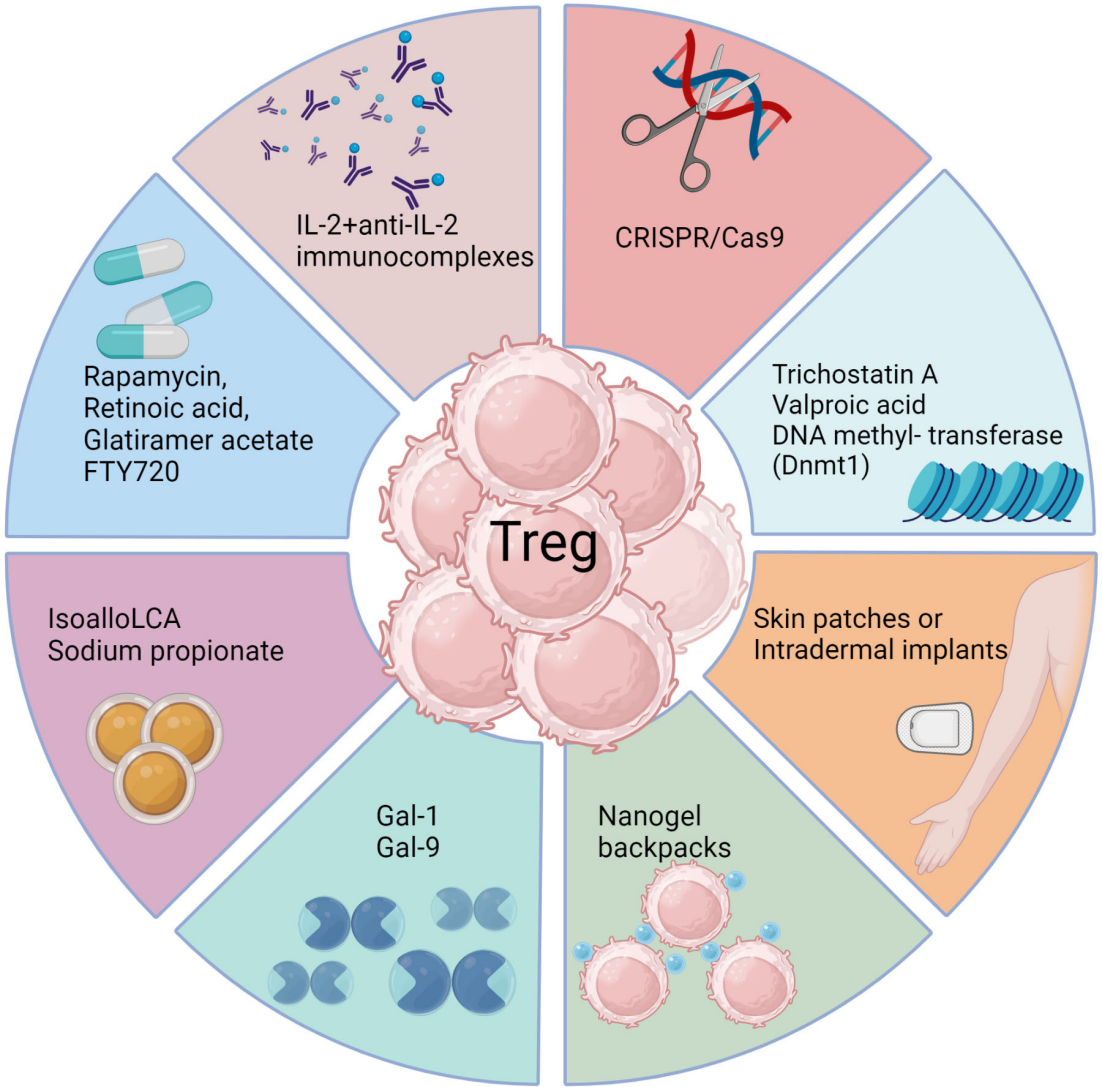
Approaches to enhance Treg responses. Several biological methods as well as pharmacological agents have been documented to selectively expand Treg cells in vivo either by achieving de novo conversion or promoting function of already committed Treg, particularly in model systems. These include use of monoclonal antibodies targeting IL-2, CRISPR gene editing, epigenetic modifiers, interleukins, galectins, bile acid metabolites, and metabolism-acting immunotherapy drugs. Each approach aims to promote Treg expansion, conversion or stability through unique mechanisms such as acting on FoxP3 enhancer the conserved non coding sequence 3 to improve Treg differentiation (isoallo-LCA bile acid metabolite), epigenetic modification and stable induction of FoxP3 (Azacytidine), proliferation and conversion (IL-2 and anti-IL-2 complexes), proliferation and enhanced suppressive activity (Rapamycin), nanogel backpack containing IL-2FC conjugated to CD45 where IL-2 is released only after Treg activation, genetically modified Treg expressing IL-10, IL-35 or absent regulators of FoxP3. These strategies offer potential ways to enhance Treg populations and rebalance immune responses in viral diseases.

There have been some drug and biological approaches described that do succeed in preferentially expanding Treg in vivo, at least in model systems (**Figure 3**) (161, 162). For example, administering the galectin molecules, such as Galectin-1 or Galectin-9, expands Treg for reasons that remain unclear. Our group used this approach to diminish the severity of herpetic ocular lesions and correlated the success with a change in the balance of T cells to increase the frequency of Treg (163). Other drugs that were reported to expand Treg include rapamycin, retinoic acid, glatiramer acetate and FTY720, but these compounds have mainly been evaluated to limit autoimmune disease lesions. They merit testing in viral model systems and perhaps also in clinical situations (161). A biological approach that caused excitement was that Treg could be expanded using immune complexes of IL-2 and mAb (clone JES6-1) to IL-2, but reports of its success to limit an inflammatory reaction caused by a viral infection are not available (164). The subset of T cells expanded by immune complexes can be critically dependent on the clone of anti-IL-2 mAb used. A different clone, S4B6, when injected in vivo expanded virus-specific CD8+ T cells in HSV infected animals and not Treg (165). In addition, complexes with clone S4B6 also promoted LCMV reactive CD8+ T cells, as demonstrated using an adoptive transfer approach (164). The paradoxical effects of the two clones of anti-IL-2 antibodies complexed with the cytokine were explained by structural analysis. While the cytokine in complex with the S4B6 clone preferentially binds to IL-2Rβ and IL-2Rγ that are predominantly present on effector cells, the JES6-1 clone complexed with IL-2 becomes dissociated from the complex facilitating the interaction of IL-2 with all the subunits of IL-2R, including the high affinity α-chain (162). As Treg preferentially express α-chain of the receptor, the cytokine is utilized by such cells more efficiently with the complexes increasing the bioavailability of IL-2 (162).

Another promising biological approach to induce Treg was a slow release of antigen delivered via osmotic pumps, which had the advantage of inducing antigen-specific Treg (166). Such an approach could be more clinically relevant with a more acceptable delivery system. Conceivably, incorporating antigens in skin patches or intradermal implants to efficiently expand Treg of a required antigen specificity could be an effective strategy, but this needs to be evaluated. Chimeric antigen receptor expressing Treg, as can be obtained by genome editing techniques such as the use of CRISPR/Cas9 (167), may also merit a trial. Further modification of such Treg to stably express transcription factors such as FoxP3 and the high affinity IL-2 receptor α-chain as constitutive modules to enhance their suppressive function represents a potential strategy to mitigate viral induced immunopathology. However, enthusiasm and application of Treg expanding approaches seems to have waned because of the plastic nature of the expanded cells. Whether a cell exhibits plasticity and can change its phenotype is largely governed by epigenetic modification (168). Therefore, drugs such as trichostatin A, valproic acid or DNA methyl transferase such as Dnmt1 that can modify the epigenome of differentiating or already committed cells could be useful (169). Such chemicals not only promote the de novo conversion of non-Treg into Treg, but also stabilize the expression of FoxP3 in already committed cells. However, selectivity and specificity are always challenges to meet with several such approaches and the epigenetic modifiers being no exception.

The composition of the milieu in which APCs and T cells engage, as well as how the antigen is delivered to APCs, could result in the induction of either pro- or anti-inflammatory cells. The composition of the milieu could be altered by several means such as the inclusion of neutralizing antibodies against pro-inflammatory cytokines, injecting anti-inflammatory cytokines such as IL-10, TGF-β or some of the immunosuppressive chemicals such as rapamycin, dexamethasone to induce tolerogenic APCs with intact antigen-presentation capability to expand or induce Treg of different types (170). The DC therapy approach could also be used wherein the cells isolated either from PBMCs or bone marrow of patients are exposed to such reagents in the presence of antigens ex-vivo. The ex vivo primed APCs are then transferred back in the patient to expand antigen specific Treg  (171). Nano formulations of organic material such as liposomes, polymers including poly lactic-co-glycolic acid, polylactide, poly(β‐amino esters), polyethylene glycol also could be generated to incorporate antigens for delivery to the APCs under tolerogenic conditions (171). For example, nanotechnology-based drug delivery approaches offer potential for stimulating or suppressing immune cell responses. Thus, functionalized carbon nanotubes activate DC by triggering TLR7 signaling pathways that lead to pro-inflammatory cytokine production and increased co-stimulatory molecule expression (172). On the other hand, native cellulose nanofibrils induce immune tolerance in DC by possibly interacting with CD209 and actin filaments, leading to altered T cell responses characterized by a weaker Th1 and Th17 response, but a stronger Th2 and regulatory T cell response (173). A novel cellulose nanofibril-reinforced hydrogel developed by Yang et al. (174) uses a pH-responsive drug release system, which may involve interactions with immune cells in the wound healing process. Additionally, Tomić et al. (175) reported that functionalized cellulose nanofibrils induce tolerogenic properties in DC, which may suppress allogeneic T cell proliferation through mechanisms yet to be fully specified. These findings demonstrate the potential of nanotechnology to manipulate immune responses through targeted drug delivery, offering new therapeutic avenues for immune-related conditions and may be beneficial to alleviate tissue damage after viral infection.

The injection of antibodies against pro-inflammatory cytokines such as IL-6, TNF-α, IL-17 are routinely used to manage inflammatory diseases (176), but the cost involved with such regimens represents a major prohibitive step. Approaches wherein the replicating microbes, preferably commensal bacteria, if modified to express and secrete such biologicals in situ could reduce the expense significantly. In fact, the feasibility of such approaches has been demonstrated, although not in a viral disease (177). More recently, a modified strain of *E. coli* was engineered to secrete a nanobody against the cytokine TNFα to dampen inflammatory response in the gut. With the neutralized pro-inflammatory cytokines regulatory mechanism could operate effectively (178). Identifying such microbes, the ease of their manipulation, the disease condition being targeted, and the regulatory compliances would all need to be factored in, should such approaches be pursued in a practical situation. The approach nonetheless opens new avenues to produce neutralizing antibodies not only against the cytokines, but obviates the need to inject purified antibodies that are expensive and challenging to employ in clinical situations.

In addition to employing cytokine neutralizing antibodies, some of the host’s metabolites could shift the balance from pro-inflammatory T cells such as Th1 or Th17 towards Treg (179). For example, bile acid metabolites. such as the derivatives of lithocholic acid (LCA), 3-oxoLCA and isoalloLCA, reciprocally regulate the differentiation of Treg and Th17 cells and when administered to mice served to reduce pro-inflammatory Th17 cells, but increased Treg representation (179). Similarly, short chain fatty acids, such as sodium propionate, helped to resolve ocular lesion caused by HSV potentially by affecting several cell types of innate as well as adaptive immune participants (155). In animals fed sodium propionate, Treg outnumbered T effectors (155). Approaches that modify cellular metabolism are cost effective, easy to apply and therefore could have translational value as is discussed in a later section.

Apart from Treg, other regulatory mechanisms, such as myeloid derived suppressor cells (MDSCs), also exhibit potent suppressive activity. Whether or not the MDSCs are induced during the early phase of a virus infection could help decide the pathogenesis of certain viral infections such as LCMV in mice. Infection with clone 13 of LCMV, that activates and expands MDSCs early after infection, results in chronic infection while the Armstrong strain of LCMV fails to efficiently signal MDSCs and the infection resolves favorably (180). Furthermore, depletion of MDSCs generated efficient anti-viral CD8+ T cell response in clone 13 infected animals, although these maneuvers had to be done at the initiation stage of infection. Other infections such as HIV and HSV can also activate and expand MDSCs early after infection (180). For example, our group showed that therapy with ex vivo differentiated MDSCs in the presence of cytokines such as IL-6, IL-4 and GM-CSF controlled the severity of herpetic ocular lesions when using a therapeutic design (181). Conceivably, strategies to expand MDSCs in vivo could alleviate inflammatory response by their direct action of effectors, as well as by expanding endogenous Treg responses.

In conclusion, rebalancing inflammatory reactions to expand regulatory mechanisms and inhibiting pro-inflammatory components represents a major objective to minimize the consequences of any viral immunoinflammatory process. There are many different forms of regulation and accessible means to expand them and dampen lesions at least in model systems. However, few if any are ready for routine use in the clinic and replace or support, for example, the use of anti-inflammatory drugs. For the long term control of persistent chronic lesions conceivably there could be a place for vaccines based on the mRNA format successfully used in COVID-19 vaccines (182). For example, this format could be designed to induce regulatory mediators and could also include the mRNA sequences of viral epitopes that expand viral specific Treg. Such an approach merits evaluation and conceivable it might become a practical procedure to achieve the rebalance of Treg and pro-inflammatory T cells that we advocate is needed to manage some chronic virus-induced inflammatory lesions.

## Rebalancing inflammatory reactions by restoring lost effector cell functions

T cell activation depends on signals received from engagement of their T cell receptors, signals from additional receptors binding to costimulatory molecules such as the CD80/86 ligands on antigen presenting cells, as well as signals from cytokines such as IL-2. Excessive activation of T cells is avoided by signaling induced by inhibitory receptors for several molecules that include CTLA-4, PD-1, TIM-3, LAG-3 and some others. In some circumstances, the activity of the inhibitory receptors becomes predominant and this serves to impair the protective function of T cells. The effect happens in several cancers, but also occurs in many chronic viral infections as was first discovered in the LCMV model of chronic infection (183) and is now referred to as immune exhaustion (184). Fortunately, oftentimes, the protective function of T cells can be restored by administering mAb that block the function of one or more inhibitory receptors and this checkpoint blockade therapy has become a valuable strategy to control some cancers (185). There is abundant evidence that the T cell exhaustion phenotype can be demonstrated in several human chronic viral infections, which infers that checkpoint blockade could be a valuable means to control such infections, although this has not been formally demonstrated in a clinical situation (186). During immune exhaustion, a gradual increase in the expression of multiple inhibitory receptors occurs and the T cells lose functions such as the production or loss of IFN-γ, TNF-α, IL-2 and compromised ability to control model chronic virus infections. The actual mechanisms involved in immune exhaustion have been a topic of intensive study using model systems and it is expected this will translate to therapeutic use in the clinic. For example, it is now clear that exhausted CD8 T cells (Tex) may consist of two subpopulations-progenitor: Tex which are CXCR5+TCF-1+PD1^Int^ and terminal Tex that are CXCR5-TCF-1-PD1^hi^. The CXCR5+ progenitor Tex shared transcriptional signatures with memory precursor CD8 T cells and hematopoietic stem cell early progenitors, while the CXCR5- terminal Tex shared transcriptional signatures with CD8 terminal effectors and hematopoietic stem cell mature cells (118). Of relevance, PD-1 blockade treatment acted on CXCR5+ progenitor Tex, which underwent vigorous proliferation, produced cytokines and conferred therapeutic benefit of PD-1 blockade therapy to clear chronic LCMV infection. On the contrary, terminal Tex did not respond to PD-1 blockade therapy (118). Furthermore, adoptive transfer of CXCR5+ progenitor Tex, but not CXCR5- terminal Tex, was effective in controlling chronic LCMV infection. Thus, approaches that target CXCR5+ progenitor Tex may be more relevant to control chronic virus infections. Additionally, it has also been observed that blocking simultaneously more than one inhibitory receptor mechanism is more effective than single checkpoint inhibitor blockade (187). For example, combinatorial blockade of PD-1 and Tim-3 was synergistic to curtail viremia during chronic LCMV infection (119). In addition, combining checkpoint blockade with other therapies may also achieve greater success than single therapy. For example, combining PD-1 blockade therapy with the provision of IL-2 in chronic LCMV was synergistic and acted to reverse CD8 T cell exhaustion by acting primarily on CXCR5+ progenitor Tex (120, 121). Similarly, inhibiting signaling by the co-stimulator molecule 4-1BB along with PD-1 led to potent suppression of viremia and more effective control of after chronic LCMV infection (122).

Although the majority of studies on immune exhaustion focus on CD8+ T cells other cell types also are subject to immune exhaustion. These include CD4 T cells (188) and NK cells (189). As regards the latter, PD-1 expression was increased on NK cells from HIV infected persons (190) some of which had Kaposi sarcoma (191). Furthermore, Tim-3 was upregulated on NK cells from patients with chronic hepatitis B virus infection (192). However, in chronic infections the outcome of receptor blockade therapy has not been assessed for effects on NK cell function, but such therapy has been recorded in some tumor systems with restored NK cell activity correlating with improved tumor control (193).

Overall, we are optimistic that checkpoint blockade will be used in the clinic to facilitate the control of some chronic viral lesions, although more research is needed to find the optimal strategies to use. Obvious candidates are chronic liver pathology caused by hepatitis viruses, particularly those caused by HBV where immune exhaustion is known to occur (194) and effective antiviral drugs are lacking such as are available to control hepatitis caused by HCV (186, 195).

## Rebalancing reactions by changing the microRNA environment

MicroRNAs (miRNAs) are small noncoding gene sequences that exist in cells and are also found in many viruses. They are usually 20-22 nucleotides in length and act to silence mRNAs and mediate post-transcriptional regulation of gene expression (196). There are an estimated 2300 different miRNAs in human cells (197), and these influence a wide range of genes that control the biological activity of cells that includes those that react to virus infections. Additionally, it is known that several viruses also encode one or more miRNAs and these too contribute to viral functions and also can affect the pathogenesis of infection (198). Accordingly, during a virus infection changes in expression levels of several miRNA species may occur many of which act to affect the function of one or more cells of the innate and adaptive immune systems that respond to the infection. Moreover, it has become evident that manipulating the expression levels of one or more miRNAs, primarily host miRNAs and usually before or early after infection, can be a useful approach to change the outcome, such as minimizing tissue-damaging consequences.

MicroRNAs can act directly or indirectly to affect the ability of a virus to replicate and the extent of tissue damage that results from the infection. Some host miRNAs are known to influence viral gene translation and replication events as well as essential steps in viral infection, such as the expression of viral receptors. Other host miRNAs may also influence the nature of the host reaction made to the infection, which is of particular relevance in chronic viral infections. This begs the question of if manipulating the expression levels of one or more miRNAs might be a practical therapeutic maneuver to minimize the extent of tissue damage caused by inflammatory reactions to viral infections, with this effect explained by a rebalanced immune reaction. Several reports have described the consequences of changing the expression levels of usually a single microRNA either by increasing levels using synthetic mimics, or reducing its presence by gene knockout or using specific antagomirs (109, 199–201). Some of the more spectacular results were obtained by manipulating miR-122 levels, a molecule expressed predominantly in hepatic tissues and necessary for the replication of HCV in the liver. In a chimpanzee model, it was shown that blocking miR122 with a locked nucleic acid-modified DNA phosphorothionate antisense oligonucleotide provided long lasting protection against their chronic HCV infection (202). This approach was subsequently found safe and reduced HCV RNA levels in humans (203), but highly effective direct antiviral drugs are now preferred to control HCV. Moreover, with the miR122 blocking studies it was not clear if the favorable outcome correlated with a rebalanced immune response pattern since such studies were not performed.

Many miRNAs affect the functions of immune cells (see **Table 4**) and changing the expression of such miRNA can result in diminished lesions. For example, studies were done showing that modulating miR155, a molecule that affects several aspects of the inflammatory reaction, may change the severity of lesions. An early study from the Baltimore laboratory showed that stopping miR155 expression using gene knockout resulted in protection from the induction of an autoimmune lesion in mice (204). This outcome was shown to correlate with a change in the pattern of immune responsiveness with less induction of lesion producing pro-inflammatory Th-17 and Th-1 cell subsets (204). Another group also showed that silencing miR-155 led to less severe clinical consequences of experimental autoimmune encephalomyelitis (EAE) (110). A similar change in outcome was noted when comparing the extent of immunopathological damage to the eyes of mice caused by HSV infection. Thus mice unable to produce miRNA, because of gene knockout or miRNA 155 blocked in normal mice with specific antagomirs, resulted in less severe ocular lesions, an effect that correlated with a diminished pro-inflammatory CD4 T cell response (109). Unfortunately, however, the treated host was left more susceptible to other complications since the virus usually disseminated to the brain causing encephalitis and death indicating the potential downside of manipulating a microRNA with likely multiple targets of action (200). It would have been of interest to evaluate if local blunting of mR-155 expression in only the eye could have achieved effective therapy.

**Table 4:** Some miRNAs that affect function of immune cells.

Targeted miRNA	Target cell	Targeting strategy	Effects on immune cells and their functions	Ref
miR155	Th1 and Th17	mir155-/- gene KO mice	miR-155 is required for development of Th17 cell and Th1 cell subsets in EAE.	(204)
			Mir-155 promotes generation of both Th1 and Th17 effector cells and increase susceptibility to EAE	(110)
		Antagomir-155 nanoparticles	Th1 and TH17 cell response diminished in HSV infected mice and reduced inflammatory eye lesion severity post miR-155 blockade	(109)
	Broncho-alveolar lavage fluid (BALF) containing macrophages and neutrophils	miR155-/- gene KO mice	Flu virus infected miR155-/- KO mice had 4-fold less IFN-γ than WT in lung wash samples taken at day 6 pi.	(205)
	B lymphocytes	miR155-/- gene KO mice	Absence of miR-155 led to compromised antibody responses	(206)
	CD8 T cells	miR155-/- gene KO mice	Absence compromises effector CD8 T cell response, antiviral response	(207, 208)
	NK cells	Lentivirus mediated overexpression and knockdown	Overexpression of miR-155 increased IFN-γ production. Conversely, knockdown decreased IFN-γ production.	(209)
miR-31	CD8 T cells	mIR-31 -/- gene KO mice	Mice lacking miR-31 had more polyfunctional IFN-γ and TNF expressing CD8 T cells than WT mice in chronic LCMV infection.	(210)
miR-29a		LCMV-specific CD8 T cells transduced with mIR-29a expressing retrovirus	mIR-29a expression attenuates CD8 T cell exhaustion and promote memory –like CD8-T cells in chronic LCMV infection.	(211)
miR-17-92 cluster		CD8 T cells in transgenic mice expressing miR-17-92 (miR-17-92/Pmel-Tg mice).	CD8+ T cells had higher cytotoxicity and IFN-γ production against glioblastoma	(212)
	Follicular helper CD4 T cells	Mice with no miR-27**∼**92 in CD4 T cells	Fewer splenic TFH cells, required for TFH differentiation	(213)
miR-15a/16	Treg	Transfection and transduction of miRNA`s into cord blood derived Treg.	Overexpression of miR15a/16 reduced the suppressive functions of Treg cells, leading to decreased expression of FoxP3/CTLA4 proteins and diminished Treg suppressive function.	(214)
		Knockdown of miR15a and miR16 in conventional cord blood T cells	Stimulated the expression of FoxP3 and CTLA4.	
miR-138	nTreg	nTreg were collected form healthy people and transfected with mIR-138	CTLA-4 and PD-1 immune checkpoint protein expressions were reduced.	(215)
	CD4+ T cells	CD4+ T cells transfected with miR-138	CTLA-4 and PD-1 immune check proteins and FoxP3 expression had downregulated.	
miR-146a	Treg	Bone marrow chimeras	Absence of miR-146a in Tregs resulted in fatal Th-1 mediated inflammation.	(216)
	CD4+ and CD8+ T cells	Mice with miR-146a absence in T cells	Deficiency of miR-146a made T cell hyper aggressive leading to autoimmunity.	(217)
	Macrophages	Overexpression	Overexpression promoted polarization towards M2 phenotype.	(218)
miR-21	Macrophages	Overexpression, knockdown	Overexpression of miR-21 increased IL-10 production. Knockdown had the opposite effect.	(219)
	Th17 cells	Mice deficient in miR-21, anti miR-21 sequences	Deficiency of miR-21 blocked Th17 differentiation, Blockade of miR-21 with antagomirs attenuated EAE in mice.	(220)
	B and T lymphocytes	silencing of miR-21 using LNA	Silencing of miR-21 mitigated splenomegaly in lupus in mice.	(221)
	B lymphocytes	Silencing of miR-21 using antisense sequences	Silencing of miR-21 increased representation of IL-10+ Breg cells and mitigated EAE disease.	(222)
miR-486-5p	NK cells	NK cells isolated from HCC patients and in-vitro transfected with miR-486-5p	miR-486-5p transfected NK showed enhanced cell cytotoxicity via activation in natural killer group 2D (NKG2D) and perforin expression.	(223)

Studies in mice have also shown that miR-155 is involved in the inflammatory reaction to FLU infection with lung injury being diminished in miR-155 knockout mice (205). Other studies with model systems have shown a critical role of a particular microRNA. For example, in chronic LCMV miR-31 plays an influential role in chronic lesions. Mechanistically, it was found that miR-31 targeted Ppp6C, a negative regulator of IFN signaling, resulting in increased levels of checkpoint molecules such as PD-1 and consequent T cell dysfunction (210). This could mean that targeting miR-31 may provide a therapeutic strategy to rebalance the pattern of immunity to control chronic viral infections. There are other studies which show enforced expression of miR-29a acts to counter CD8 T cell exhaustion and improved CD8 T cell function implying that upregulating miR-29a may be another approach to diminish the consequences of some chronic viral infections (211). It also could be that microRNA manipulation could be useful to influence some critical steps in viral pathogenesis one of which could be angiogenesis. Thus the angiogenesis process is influenced by several microRNAs (224). Our group could show that suppressing miR-132, which influences signaling by the angiogenic factor VEGF, led to diminished ocular lesions caused by HSV (225). Unfortunately, to achieve success against pathological angiogenesis, especially in the eye, requires therapy during the development of pathological angiogenesis since once present, as occurs in chronic lesions, its removal is highly problematic.

Perhaps of no surprise several studies have been performed to record the role that microRNAs could be playing in COVID-19 pathogenesis. Several host microRNAs show changed expression (226, 227), but of interest would be if microRNA manipulation therapy would be of value to help control the inflammatory stage of COVID-19 infection. To this issue, one report showed that exosomes containing miR-145 and miR-885 regulate thrombotic events in COVID-19 patients (228). In addition, animal models of infection have indicated that miR-155 inhibition can control the inflammatory effects of COVID-19 (229), as we mentioned is also the case in other viral immunopathologies. We anticipate that the future will see more studies evaluating how manipulating one or more microRNAs will help control COVID-19 infection.

In conclusion we discussed examples where changing singular microRNAs was effective at changing the outcome of inflammatory viral infections. Examples of success are few and are largely confined to model systems. However, we are optimistic that the field is worthy of more investigation and also should consider evaluating multiple microRNA changing cocktails that target different aspects of viral pathogenesis and also perhaps combining microRNA manipulation with other approaches that succeed in rebalancing the nature of immune responses to infections.

## Rebalancing reactions by targeting metabolic pathway differences in inflammatory lesion participants

**Figure 4:**
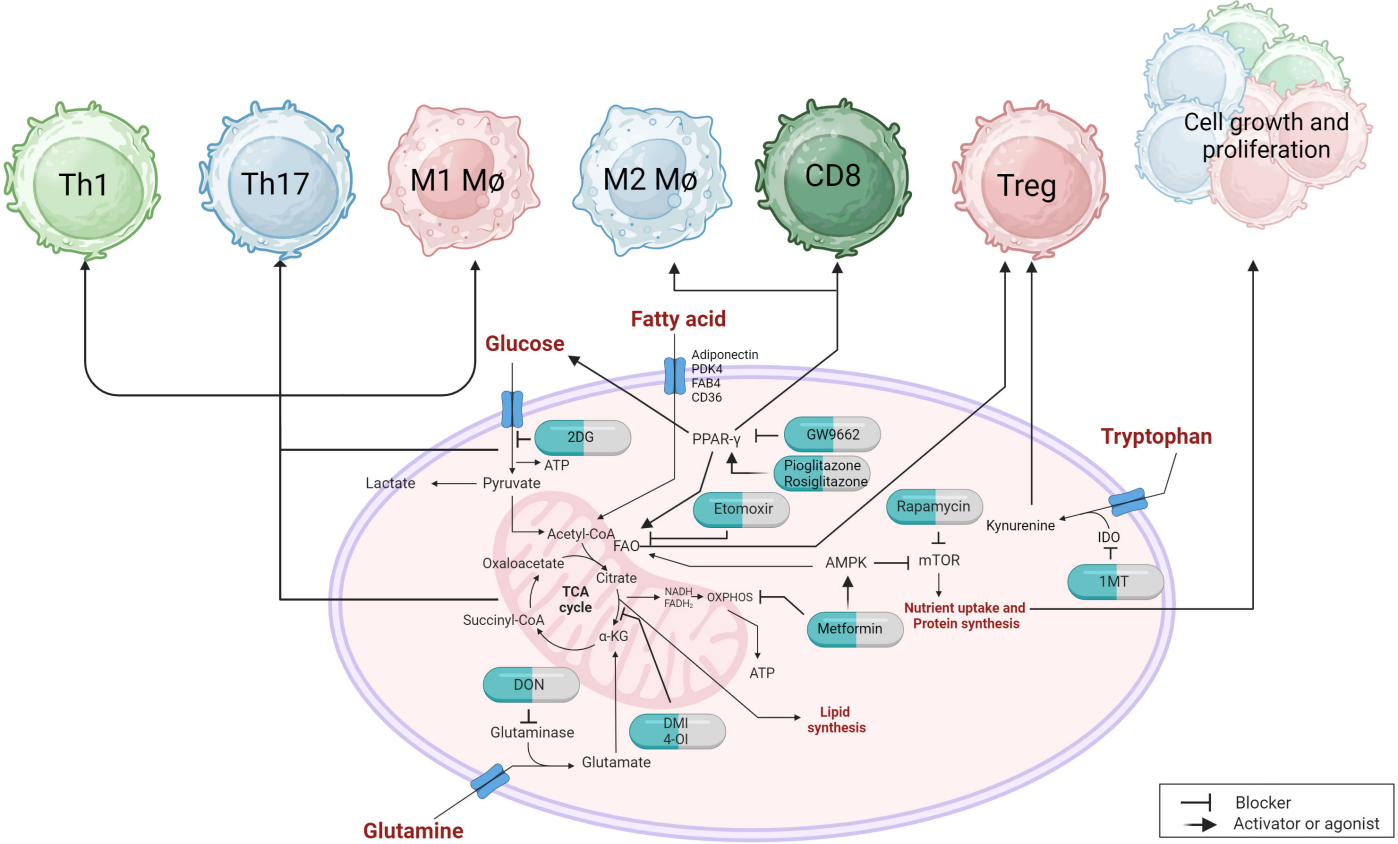
Metabolic targets and immunotherapeutic approaches can have an impact on immune responses. Targeting the glycolytic pathway with 2DG can influence the proinflammatory response of immune cells, including Th1, Th17, and M1 macrophages. PPARγ, which regulates fatty acid metabolism, can also modulate glucose uptake and glycolysis. The use of agonists or inhibitors can affect M2 macrophages and CD8 T cell responses. PPARγ also plays a role in Treg that utilize fatty acid oxidation (FAO). Inhibiting carnitine palmitoyltransferase 1 (CPT1) with etomoxir prevents the transport of long-chain fatty acids into the mitochondria, thereby impairing the suppressive capabilities of Treg cells. The AMPK activator metformin promotes FAO while inhibiting oxidative phosphorylation (OXPHOS) through the inhibition of complex chain I, which can restore immunosuppressive functions and reduce proinflammatory cell proliferation by indirectly inhibiting mTOR. Inhibitors of glutaminase, such as DON, or derivatives of itaconic acid like DMI and 4-O1, interfere with the tricarboxylic acid (TCA) cycle and can alleviate proinflammatory cell responses. The uptake of tryptophan, which directly affects the suppressive functions of Treg, can be reduced by an inhibitor called 1MT, which inhibits the IDO enzyme.

During the course of any virus infection both the cells infected by the virus and the host cells that respond to the infection undergo metabolic reprogramming to support the infection and to influence its outcome, respectively. When a virus infects a cell, several metabolic changes usually occur before new virions are produced and modifying these changes provides an approach to reshape the impact of the infection (230). In the current review, we are focusing on viral infections where lesions are mainly the consequence of a host inflammatory response to the infection raising the question of if modulating one or more metabolic pathways represents a practical approach to limit the extent of lesion expression. The multiple cell types that respond to infection may show different metabolic signatures with respect to the various pathways that are mainly reprogrammed. This opens up the prospect of targeting the metabolic pathways used by cells that are more tissue-damaging with control measures that will suppress their activity, or block their induction (**Figure 4**). Oftentimes, the main tissue damage is mediated by activated subsets of T cells, such as Th1 and Th17 cells, or M1 macrophages, which mainly metabolize glucose via the glycolysis pathway, which rapidly supplies their energy needs (231). Other cell types in the inflammatory reaction, such as Treg that can limit the extent of tissue damage, may derive their energy mainly from alternative pathways such as fatty acid oxidation and oxidative phosphorylation (OXPHOS) (232). In consequence, using drugs that target glycolysis can blunt the participation of pro-inflammatory cells and preserve the regulators, thus limiting the extent of tissue damage. This strategy of manipulating metabolic pathways to rebalance inflammatory reactions has been explored mostly to control autoimmune lesions and some cancers, but the approach has been evaluated more recently with some chronic viral induced lesions, as reviewed by us recently (233).

Basically, there are two major strategies that could be used to manipulate metabolic events to counteract tissue damage caused by a viral infection. One approach is to inhibit critical metabolic pathways in the cells directly involved in mediating tissue damage, so disarming their ability to cause the damage (234). The other approach is to use metabolic modulators either before or during the development of the inflammatory reactions with the objective of minimizing the induction of immune components that cause the tissue damage, but at the same time retaining the protective and regulatory aspects of the immune responses (235). Most experimental work on metabolic modification to control viral immunoinflammatory lesions have used the latter approach.

The objective to diminish the extent of ongoing viral reactive inflammatory reactions is usually achieved with a range of anti-inflammatory drugs such as steroids and other anti-inflammatory reagents, which do not target any particular metabolic pathway (236). It is often the case that in active immunoinflammatory lesions, the cells that orchestrate and participate in lesions are some subsets of T cells and M1 type macrophages that are activated and need to obtain an immediate source of energy, which is mainly supplied by metabolizing glucose via the glycolysis pathway (234). Accordingly, the use of drugs such as 2-deoxy-d-glucose (2DG), which cannot be further metabolized by downstream enzymes in the glycolytic pathway, could be merited. However, when 2DG has been used to inhibit viral immunoinflammatory lesions, it has usually been to control the development of inflammatory cell induction and mediator production rather than used as a treatment therapy (114).

There are potential approaches to achieve inflammation disarming therapy, one of which could be to target the AMPK mediated mTOR pathway that acts as a metabolic regulator during inflammatory cytokine induction, such as IFN-γ, TNF-α and some chemokines (237). The drug rapamycin could achieve this effect, as has been shown in the case of some autoimmune lesions and cancers (238). A prior study from our group showed that rapamycin administration markedly diminished the severity of herpetic ocular immunoinflammatory lesions, but did not evaluate if rapamycin therapy could diminish the severity of already established lesions (239). There are also reports that the drug metformin, which acts primarily to inhibit energy metabolism via the OXPHOS pathway in mitochondria and likely the activation of AMPK, can be useful to attenuate the severity of some autoimmune disease lesions (240). Moreover, unconfirmed reports claim that metformin can diminish the severity of inflammatory lesions caused by COVID-19 (241). In line with this, some patients after COVID-19 infection that suffer with the troublesome syndrome Long-COVID have benefitted by therapy with metformin (242).

Other approaches worth exploring to disarm inflammatory cells during viral immunoinflammatory lesions include using drugs such as GW9662 that modulates PPAR-γ, which is involved in regulating glucose and lipid metabolism as well as was mentioned previously some genes involved with inflammation (243). The metabolism relevant genes include FAB4, CD36, adiponectin (responsible for lipid uptake), FASN (lipid synthesis), GLUT4 and pyruvate dehydrogenase kinase 4, which are responsible for glucose metabolism and fatty acid oxidation. When PPAR-γ is activated, it promotes the uptake and storage of fatty acids and glucose in immune cells, such as macrophages, leading to a shift towards the M2 IL-10 producing anti-inflammatory phenotype (243). Conceivably, the upregulation of PPAR-γ, as can be achieved with agonist drug therapy, may result in diminished lesions. Such an effect was reported in the case of lung inflammatory lesions caused by the 2009 H1N1 pandemic FLU virus (244) indicating that the approach should be explored to disarm other viral inflammatory lesions.

The majority of observations supporting the notion that manipulating some aspect of metabolism can rebalance immune response patterns and alleviate the severity of viral immunopathology have observed the effects of changing the metabolic climate either before or early during the development of the viral tissue-damaging events. For example, there are many situations where the absence for genetic, dietary or therapeutic reasons of some metabolic activity may result in changing the outcome of a virus infection. A contemporary example came from the recent COVID-19 pandemic where it was well documented that those with diabetes and significant obesity suffered more severe immunoinflammatory lung lesions and often succumbed to the infection (245). Many patients were kept alive by using anti-inflammatory drugs and mAb against inflammatory cytokines. In addition, the frequent sequel to COVID-19 infection, Long-COVID, is suspected to be at least in part a metabolic problem, although its nature remains ill-defined and metabolic reprogramming is not currently used as a treatment modality (242).

Other metabolic changes that affect the expression of a viral infection include problems with tryptophan metabolism. Thus, if the essential amino acid tryptophan is depleted for some reason, one of its metabolites, kynurenine, accumulates and this has immunosuppressive effects on immune control (246). One means by which tryptophan can be depleted is that the enzyme indoleamine 2,3-dioxygenase (IDO), that is induced by some viral infections, catalyzes the breakdown of tryptophan into kynurenine, leading to tryptophan depletion, kynurenine accumulation and suppressed immunity (247). Therefore, inhibiting the activity of IDO, or replenishing tryptophan, may be a therapeutic strategy for controlling the initial phases of a viral infection as has been shown in experimental FLU virus infection (248). Curiously, animals unable to produce IDO, because of gene knockout or drug suppression, do exhibit less severe immunoinflammatory lesions in experimental FLU and RSV infections (248), but it is at yet unclear if manipulating tryptophan metabolism would succeed in suppressing already established viral inflammatory lesions.

Diet affects metabolism in several ways and diet can impact on the response pattern to an infectious disease. Thus, in our own studies we could show that supplementing the diet with short chain fatty acids such as propionate (155) and butyrate could diminish the severity of the ocular inflammatory response to HSV explained by a change in the ratio of pro-inflammatory and regulatory T cells to favor the latter in lesions. Similarly, the studies on FLU by Trompette et al. showed that feeding mice butyrate as a dietary supplement increased their resistance, seemingly because the balance of their immune reactivity was shifted to favor a superior protective CD8 T cell response (249). In other viral systems, the composition of the diet can also influence the pathogenesis of infection. One favored model has been to compare the outcome of viral infections in animals fed high fat or low fat diets. For example, in a study of mice infected with H1N1 FLU virus those fed a high-fat diet (HFD), which induced obesity, developed more severe inflammatory lung disease, higher levels of inflammatory cytokines, and higher mortality than those fed low-fat diets (250). Another group also showed that HFD led to increased levels of ROS and myeloperoxidase (an enzyme that indicates neutrophil activation) in lung homogenates compared to low-fat diet groups, an effect they correlated in part with a reduced NK cell response in HFD recipients (251). Other groups have linked the greater susceptibility of a HFD to an increased inflammatory neutrophil response, which can increase in number by as much as 20-fold (252). One of the consequences of feeding certain diets such as unsaturated fats and high calories is that the adipose tissue may become pro-inflammatory and produce cytokines such as GM-CSF, IFN-γ and granzyme B, which in turn disrupts the balance of T cell induction contributing to tissue-damaging lesions that occur during chronic inflammation (253).

There is a strong suspicion that HFD and obesity are risk factors in humans for both severe FLU and COVID-19 infection, with the underlying mechanism related to dysregulation of the immune response and a delay in tissue healing and recovery, but further mechanistic studies are warranted to establish how these effects are mediated. There are also suggestions that many dietary supplements, such as amino acids, vitamins, minerals, and omega-3 fatty acids, may be able to support effective immune functions and potentially reduce the severity of viral infections. However, clinical trials in this area are limited, and more research is needed to confirm the many claims that are made. There does appear to be a strong case that Vitamin A (VitA), which is necessary for immune cells such as T and B lymphocytes to function normally, is useful (254) and some advocate VitA supplements for measles infection to reduce its severity and the duration of measles-related symptoms. A similar consequence was advocated to apply to COVID-19 (255), but further evidence is still needed. Other studies have shown that diets supplemented with nutrients such as L-glutamine, vitamin C, omega-3 fatty acid derivatives or zinc may all improve the outcome in COVID-19 patients (256–259). It is not clear, however, if supplementing diets with glutamine is always a useful approach to control viral inflammation. Thus, two groups showed that suppressing glutamine with the inhibitor 6-Diazo-5-oxo-l-norleucine attenuated viral immunoinflammatory lesions caused by HSV and Sindbis virus infections (260, 261). These observations correlated with a marked reduction in the pro-inflammatory T cell response to the infections.

Some of the more convincing data showing that manipulating metabolic events is a valuable approach to rebalance the pattern of an inflammatory response to virus infection was done by using drugs that change metabolic events when given during the course of an infection. For example, Varanasi K. et al. showed that inhibition of glucose metabolism with 2DG administered in the early stage of HSV ocular infection markedly inhibited the immunoinflammatory lesions of stromal keratitis (114). The beneficial outcome correlated with a rebalance of cell type representation in lesions with pro-inflammatory T cells markedly reduced in numbers, but Treg were unaffected and hence became dominant in lesions. Accordingly, 2DG therapy was a valuable approach to control an immunoinflammatory viral lesion and acted by rebalancing the response pattern. However, using 2DG to control viral immunoinflammatory lesions can result in complications as observed initially by the Medzhitov and coworkers using a FLU virus model in mice (262). In this instance administering 2DG to FLU infected mice resulted in mortality. Our group also showed that controlling herpetic lesions with 2DG therapy was potentially hazardous (114). Thus, HSV is a neurotropic virus with severe, often lethal consequences if virus enters the CNS causing the syndrome herpes simplex encephalitis (HSE). We could show that therapy with 2DG started when replicating virus was still present often led to HSE and a lethal outcome (114). The result appeared to be the consequence of a failure of the inflammatory reaction in the peripheral nervous system to prevent viral dissemination to the brain (263). Other drugs that affect energy metabolism were subsequently studied. These included metformin, a drug that inhibits OXPHOS in the mitochondria and etomoxir which inhibits energy metabolism derived from fatty acid oxidation. Both drugs successfully inhibited the severity of ocular lesions (264). However, neither drug caused HSE, a result explained by minimal inhibitory effects on the protective inflammatory reactions that occur in the local peripheral nerve ganglion and which confines HSV to the ganglion in the form of a latent infection (264).

There is some interest in using the molecule Itaconic acid which affects energy metabolism by inhibiting the enzyme succinate dehydrogenase, which participates in the TCA cycle. In a study on the inflammatory reaction to FLU infection in mice, itaconate and its derivatives (dimethyl-itaconate and 4-octyl-itaconate) given daily from the onset of infection reduced lung lesions and protected from death effects perhaps mediated by suppressing by IFN-γ and other inflammatory cytokines (265).

So far there are minimal studies that describe the use of metabolic reprogramming to control inflammatory viral infections in human diseases, but the approach does merit further exploration. The future for using metabolic reprogramming to control viral immunopathogenesis may be to use mixtures of drugs that affect different metabolic pathways perhaps also administering different modulator cocktails at varying stages of infection and perhaps combined too with other rebalancing approaches that were discussed.

## Conclusions

The modern world`s success with controlling viral infectious diseases has been spectacular whenever effective vaccines are available and impressive too when the virus infection can be managed successively with antiviral drugs, as occurs in the rich world with HIV and HCV. In this review, we have discussed how we might control those viral infections where the lesions are mainly the consequence of a host reactive response to the virus with the lesions often being chronic. We advocate that when such lesions do occur it is often the case that there are components of host responsiveness that are causing tissue damage, but at the same time other ongoing reactions that are anti-inflammatory and are in the process of alleviating the extent of lesions and are facilitating resolution. This should provide an opportunity to rebalance the involvement of the damaging and protective participants and minimize the impact of the infection. This raised the question of how we might achieve such an objective, particularly when faced with an ongoing chronic viral infection in the clinic. We identified and discussed six different categories of host responses that could be manipulated to achieve our objective and described examples where success has been reported. In almost instances of success, there was a caveat. Thus, most successful procedures were achieved using model animal infection systems that would be difficult, or perhaps impossible, to translate to clinical application. The second caveat was that experimental therapies more often than not perform the manipulations either before or very early after infection. However, in a practical clinical situation the problem requiring attention is usually an established lesion that needs to be counteracted. Nevertheless, progress is being made and a silver lining of the COVID-19 pandemic, where the severe pulmonary lesions represent an example of the problem we seek to solve, many otherwise experimental procedures were evaluated and shown as successful to contain inflammatory events and achieved what we would interpret to be immune rebalancing.

The first category of events discussed was the prospect of rebalancing host innate responses to infections, but few if any practical maneuvers were revealed. Thus innate influences mainly come into play during the initial stages of viral pathogenesis and rebalancing such responses during clinical lesions is highly problematic. Some succeed such as blunting the effects of inflammatory cytokines and others have been successful in model systems. Most notably the latter include destroying active macrophages with chlodronate containing liposomes, or using chemical reagents that change cells from the M1 to become M2 type macrophages These approaches, however, are not approved yet for the clinic. We suggest that other means to block cytokines and chemokines are needed one of which could be to construct mRNA vaccines to induce anti-cytokine responses, although turning off this therapy when no longer needed would be problematic.

The second and third categories of control measures discussed was to block the adaptive immune orchestrators of lesions, which are usually T cells or expand the activity of regulator mechanisms. These can be highly effective rebalancing strategies in model systems, but have yet to find much value in the clinic. Our optimism is highest for finding practical approaches that will enhance Treg responses, especially those that are antigen specific and functionally stable.

The fourth category was to describe ways to restore the function of formerly protective T cell responses that lose potency in a chronic inflammatory environment. This topic is referred to as immune checkpoint therapy and this therapy has been highly successful to inhibit some cancers. Immune exhaustion occurs in many chronic viral infections and one of the successful therapies was discovered using a chronic viral disease model (LCMV). One expects immune checkpoint therapy to find a place in the clinic to rebalance immune reactivity in a human chronic viral infection, but this has yet to happen. We are staying tuned!

The issue of achieving immune rebalance and lesion control by manipulating the expression of one or more miRNAs was the fifth topic discussed. Again an abundance of encouraging positive data has come from model animal studies, but as yet none applied to the clinic. We made the case that some aspects of viral pathogenesis may be more available for adjusting miRNAs than others with pathological angiogenesis topping our favor list. It might also be that different miRNAs need to be changed in expression during the course of viral pathogenesis and microRNA adjustment might be more effective using multiple reagents or combining miRNA manipulation with other rebalancing approaches we have mentioned.

Finally we advocated that manipulating metabolic pathways or changing metabolism by dietary changes could be a way of rebalancing immune reactivity. We described the accumulating success stories on this topic and remain highly enthusiastic about this objective. So far, however, experimentally infected mice are the main beneficiaries, but success for chronic infection control in mankind could be just around the corner.

## Author contributions

SM: Conceptualization, Data curation, Formal Analysis, Validation, Writing – original draft, Writing – review & editing. EB: Data curation, Validation, Visualization, Writing – original draft, Writing – review & editing. SS: Data curation, Writing – original draft, Writing – review & editing, Validation. BR: Conceptualization, Formal Analysis, Funding acquisition, Resources, Supervision, Validation, Writing – original draft, Writing – review & editing, Visualization.
